# 2350: The role of gut microbiota in the susceptibility of Parkinson disease development

**DOI:** 10.1017/cts.2017.43

**Published:** 2018-05-10

**Authors:** Dimitri Koutzoumis, Jose Antonio Pino, Sharonda S. Harris, Marisol Quiroz, Mansour Mohamadzadeh, Gonzalo Enrique Torres

## Abstract

OBJECTIVES/SPECIFIC AIMS: Several clinical studies have established a correlation between changes in relative bacterial populations in the gut and Parkinson disease. However, few published experiments have been able to parse out whether these associations are causative or correlative. Our aim is to determine how bacteria in the gut may impact the health and resilience of dopaminergic signaling. Our experiment is designed to serve as a proof-of-principle that controlled alterations to the gut microbiome alters mechanisms in dopamine homeostasis in the midbrain. METHODS/STUDY POPULATION: Bacterial inoculation 8–10-week-old germ-free male mice (C57BL/6) were exclusively used in this experiment. Mice were orally gavaged every 3 days (D0, 3, 6, and 9) with 100 µL novel bacterial suspension (~108 CFU resuspended in PBS with 1.5% NaHCO_3_) or vehicle and were sacrificed on D11. Tissue preparation—brains were quickly extracted and the striatum was isolated and homogenized in either RIPA buffer with protease inhibitors (for Western blot analysis) or in 0.1 N HClO_4_ (for HPLC processing). The homogenates were processed through fractional centrifugation to remove cellular debris. Lysate samples were frozen at −80°C until ready for analysis. Protein expression quantification—expression of proteins were measured using intensity of bands from Western blots. Lysates were denatured prior to loading with LB with 10% β-mercaptoethanol and 30-minute incubation at 37°C. All immunoblots were normalized to immunoreactivity to α-tubulin. Immunoblot intensity was determined using the ImageJ software. Dopamine/dopamine metabolite quantification HPLC analysis was used to determine dopamine and dopamine metabolite concentration. Aliquots of the lysate were injected onto a C18 column using a mobile phase consisting of 50 mM H_2_NaO_4_P·H_2_O, 0.72 mM sodium octyl sulfate, 75 µM Na_2_ EDTA, and 10% acetonitrile (pH 3.0). The mobile phase was pumped through the system at 0.3 mL/minute. RESULTS/ANTICIPATED RESULTS: Measured total dopamine concentration through HPLC analysis in the striatum showed no significant differences in the bacteria-treated group relative to the control group. The metabolites DOPAC and HVA had an elevated measured concentration in the bacteria-treated group relative to the control group. Western blot analysis showed decreased immunoreactivity for DAT and TH in the bacteria-treated group compared with the control group. There was no significance difference in the immunoreactivity for VMAT2. DISCUSSION/SIGNIFICANCE OF IMPACT: This study demonstrates that dopamine signaling dynamics in the midbrain can be altered by changes in the gut flora in mice. These results further substantiate the impact of the gut-brain axis and may even point to a potential avenue of bolstering the resilience of dopaminergic neurons in preventing the onset of PD. Further experiments must be performed to understand the mechanism of the observed changes and to determine if these changes have any salutary effect.

